# Study of heart rate variability in healthy humans as a function of age: considerations on the contribution of the autonomic nervous system and the role of the sinoatrial node

**DOI:** 10.3389/fmed.2025.1597299

**Published:** 2025-08-07

**Authors:** David A. Jorge Tasé, Leopoldo Garavaglia, Magdalena M. Defeo, Isabel M. Irurzun

**Affiliations:** ^1^Laboratorio de Procesamiento de Señales y Comunicaciones, Departamento de Electrónica, Facultad de Ingeniería, Universidad de Buenos Aires, Buenos Aires, Argentina; ^2^Centro de Simulación Computacional para Aplicaciones Tecnológicas (CSC-CONICET), Buenos Aires, Argentina; ^3^Centro de Investigaciones Opticas (CIOp-CCT La Plata. CONICET), La Plata, Argentina; ^4^Hospital Interzonal General de Agudos “Prof. R. Rossi”, La Plata, Argentina

**Keywords:** heart rate variability, sinoatrial node, autonomic nervous system, fibrosis, scaling, white noise

## Abstract

**Introduction:**

This study focused on the complex structure of heart rate variability (HRV) in the healthy heart. We studied the behavior of the heart rate variability (HRV) in healthy humans as a function of age from conception, including fetal data. We calculated statistical quantities such as the mean value of RR intervals (<RR>), the standard deviation of these intervals (*SD*), the power of the very low frequency (VLF), low frequency (LF) and high frequency (HF) bands, and the large-scale properties of HRV. We rationalized our findings discussing previous results that identify the signatures of the sinoatrial node (SAN) and the autonomic nervous system (ANS) in the HRV. This work provides further insights into the contribution of the SAN and the ANS to the cardiac rhythm in humans.

**Methods:**

We analyzed a total of 205 HRV time series of healthy subjects. Of these time series, 195 correspond to born individuals aged between 1 month and 74 years, 50% females. The remaining ten time series correspond to healthy fetuses. The age was expressed in weeks, including gestational age to avoid finite size effects due to rounding errors. We included results previously reported in the literature.

**Results and discussion:**

<RR> followed a power law with age from gestation and throughout life. The scale factor was 0.15 ± 0.01. *SD* evolved during pregnancy and underwent a sudden change at birth. From birth to puberty *SD* followed a scale behavior with age and a scale factor equal to 0.37 ± 0.04. The power spectrum density (PSD) was calculated as a function of age. We calculated the power of the VLF, LF, and HF bands. The HF/VLF ratio followed a power law with age, and the scale factor was −0.55 ± 0.06. The power spectrum was calculated, and we analyzed the effect of age on the scale behavior. Both the scale factor and the frequency range in which it was determined depended on age. scale behavior began at low frequencies and evolved from 1/*f*^2^-like behavior to 1/*f*-like behavior during pregnancy. After birth, 1/*f* behavior extended to occupy the entire frequency range in the puberty. With aging, the frequency range regressed again toward lower frequencies. The results demonstrated that the complex structure of HRV primarily reflects the structural complexity of the SAN, which continuously evolves from the fifth month of gestation and increases until reaching its peak at puberty. There is a white noise component in HRV that can be attributed to the ANS. Therefore, the SAN, structurally evolving from its appearance in the fifth month of gestation and throughout life, could be responsible for modulating the neuronal stimulation provided by the ANS.

## 1 Introduction

Heart rate variability (HRV) is the physiological variation in the duration of cardiac cycles. Several studies demonstrated variations in the HRV characteristic in relation to age and sex (1–12). HRV alterations were reported after myocardial infarction, in the development of congestive heart failure or in diseases leading to or accompanied by autonomic dysfunction like diabetes (13–19).

In the healthy heart two mechanisms mainly determine the HRV: the autonomic nervous system (ANS) and the sinoatrial node (SAN) (20–22). Yaniv et al. analyzed the differences in the HRV between basal and ANS blockade conditions in human and canine electrocardiograms to reveal the SAN and ANS contributions. They demonstrated that, in adults, the characteristic respiratory peak in the high-frequency (HF) band and the baroreceptor-reflex peak in the low-frequency (LF) band are completely eliminated after ANS blockade. Thus the power in the LF and HF bands is predominantly determined by the ANS. Furthermore, the main spectral contribution of the SAN is in the very low frequency (VLF) band. The SAN contributes to the large-scale (long-term) behavior of the HRV, while the ANS contributes to the short-scale region. The contribution of both the SAN and the ANS varies with age and decreases with aging.

In this work, we performed a comprehensive study of heart rate variability (HRV) in healthy humans as a function of age from conception, including fetal HRV data. We argued that this analysis may provide additional evidence to further clarify the contribution of the ANS and the SAN to the HRV. We calculated statistical quantities such as <RR> and *SD*, as well as the VLF, LF, and HF bands, and the large-scale properties of the HRV.

The human SAN has a complex structure formed by groups of specialized cardiomyocytes, with a variety of different electrophysiological profiles, tangled within connective tissue strands of collagen, elastin, and fibroblasts (23, 24). This fibrotic matrix infiltrates the SAN, determining its microstructure while providing mechanical protection and electrical insulation. The SAN was identified in human fetuses from the fifth month of gestation, and a direct correlation was established between the degree of fibrosis and age, which is also strongly correlated with a slower intrinsic heart rate and slower conduction in human and mammal hearts (25–28).

The analysis presented in this work supports the hypothesis that the structure of the SAN modulates the neural input of the ANS, and that the HRV reflects these changes. There is a white noise component in HRV that can be attributed to the ANS.

## 2 Materials and methods

We analyzed a total of 205 time series of healthy subjects available in PhysioNet: The research resource for complex physiological signals. Of these time series, 154 correspond to Irurzun et al. (12, 29), 13 to Moody (16, 30), 28 to Stein (31), 5 to Jezewski (32, 33), and 5 to Behar (34, 35).

Participants in (12, 29) were 50% females. They were aged between 1 month and 55 years. Participants in (16, 30, 31) ranged in age from 20 years to 74 years, and 50% of them were females.

In the Results the age was expressed in weeks from the moment of conception to combine the data with those obtained from fetuses and to eliminate finite size effects due to rounding of ages, i.e., those coming from the precision with which age is reported. Of course, this requires information about the pregnancy and birth of the participants. The data of Irurzun et al. (12, 29) were acquired and processed by us, and we had this information on all children under 6 years of age. As age increases the finite size effects become negligible, and the age since gestation in weeks can be estimated from the age in years and assuming a gestation of 40 weeks. The relative error in this calculation is negligible and decreases progressively with age. The age distributions in Table 1 is given as histograms dividing the population into subgroups, although we considered age as a continuous variable as in Garavaglia et al. (12).

**Table 1:** Age distribution of the 195 born individuals used in this work (29–31).

**Age range (in weeks from conception)**	**Number of patients**
41 < *x* ≤ 57	18
57 < *x* ≤ 74	28
74 < *x* ≤ 97	17
97 < *x* ≤ 140	15
140 < *x* ≤ 300	15
300 < *x* ≤ 400	27
400 < *x* ≤ 665	13
665 < *x* ≤ 1604	18
1604 < *x* ≤ 3168	21
3168 < *x* ≤ 3900	23

The 195 time series of born individuals in Table 1 are 24h long, and they were previously used and consistently compared with different population groups. Further details about participant recruitment, acquisition of electrocardiographic recordings, heartbeat annotation, artifact removal, stationarity and surrogate analysis were presented in previous works (12, 16, 29–31, 36, 37).

The remaining ten time series correspond to healthy fetuses. Five of them were obtained from fetal electrocardiogram recordings from five different women in labor, between 38 and 41 weeks of gestation (32, 33). Each recording is at least 5 min long and contains the reference fetal electrocardiogram recorded from the fetal head. The locations of the R peaks were annotated and reviewed by cardiologists. Unfortunately, the information about individual gestational age was missing. We used these series either assigning to all of them a gestational age of 40 ± 1 weeks or averaging them.

The other five time series were obtained from Behar (34, 35). This database contains a total of of 55 non-invasive fetal electrocardiogram (FECG) recordings, taken from a healthy individual between 21 and 40 weeks pregnant. The authors provided data on the gestational age for each record in weeks. The records have variable duration, and the locations of the R peaks are not annotated. We visually examined the recordings and were able to select five of them in which we could unambiguously identify the R peaks for at least 5 min. These records correspond to gestation weeks 22, 23, 25, 38, and 40. We used the software available in Behar (34) to annotate the records and extract the RR time series. In this way we avoided interruptions in the time series, since the criteria that currently exist to eliminate artifacts have not been fully tested in series from fetuses (38). We used the resulting series to determine statistical magnitudes and large-scale behavior, which appears at low frequencies where noise is negligible. We evaluated the possible influence of noise on the reported magnitudes by applying a filter based on the singular spectrum analysis (SSA) technique (39). The behavior at high frequencies in these series could not be evaluated due to the level of noise. Finally, we compared our results with other studies of HRV in healthy fetuses and showed that our data are compatible with previous information (40–42).

<RR> and *SD* were calculated using the complete series, without detrending procedure. The length of the time series essentially limits the range of frequencies that can be studied, and this fact must be considered when comparing results obtained from short and long series. The series of born individuals are stationary and sufficiently long, so that the statistical indices were independent of the series length. We assigned an error to the <RR> and *SD* values of each individual following the procedure used to evaluate stationarity. The fetal time series were at least 5 min long, artifact-free, and stationary, meeting reliability criteria developed by Mølgaard et al. (38).

The complex nature of HRV is manifested in its frequency structure, described by the dependence of the power spectrum (*S*(*f*)) on the frequency measured in 1/beat. In this work, the power spectra were calculated using fast Fourier transform (FFT) after normalizing the series to the zero mean value. To reduce noise, an averaged power spectrum was calculated following the procedure described in Andrés et al. (36) and Irurzun and Mola (37). The time series were divided into segments of 1024 points that were normalized to zero mean value. Then, the power spectra were calculated and averaged. No interpolation method was used (12).

At high frequencies, a peak attributed to respiratory sinus arrhythmia appears superimposed on a continuous background. Without considering that peak, *S*(*f*) can be modeled as a superposition of correlated signals (or the so-called colored noises). The spectral density of the correlated signals follows a power law with frequency, and the value of the scale factor determines their color. A signal is white if its spectral density is flat or has zero scale factor, so all frequencies contribute equally to the spectral density. The spectral density of the pink signals has a scale factor of -1 (1/*f*-like behavior) and that of the brown signals has a scale factor of -2 (1/*f*^2^-like behavior).

At low frequencies S(f) is written as


(1) $$\begin{array}{l} S\left(f\right)\alpha f^{-\beta } \end{array}$$

where β takes values that depend on age. The values of β were calculated in Garavaglia et al. (12), except for the HRV series of fetuses, which were calculated in this work following the same procedure The scale factor (β) is related to the α factor calculated with DFA in Garavaglia et al. (12)

An alternative analysis consists of calculating the power spectrum (or power spectral density, PSD) of a real-time series, which is constructed from the HRV of five-minute recordings by using interpolation methods (43). In healthy adult humans, three main frequency bands called high frequency (HF), low frequency (LF) and very low frequency (VLF) are considered. These frequency bands were standardized by the Task Force of the European Society of Cardiology based on the review of years of empirical evidence from various studies (44, 45) that applied frequency analysis of the HRV and observed regions of interest within the PSD. The HF band corresponds to rhythms with periods between 2.5 and 7 s (45) and is related to time domain indices such as *p*_*NN*50_ or *rMSSD*_*RR*_. The LF band corresponds to rhythm modulations with periods between 7 and 25 s and is considered to be highly influenced by the sympathetic system, but recognized to be a mixture of sympathetic and vagal modulation (46). These bands require a time series of at least 5 min as we used in this study.

The VLF band corresponds to rhythms with periods between 25 and 300 s. This band is best evaluated over 24h, because more rhythms with periods in the VLF band can appear in 24 h than in 5 min (47–49). These contributions were taken into account in our data from born individuals. The power of the VLF band in fetuses may have been underestimated, but this does not alter our conclusions. Rhythms with periods between 300s and 24h contribute to the ultra-low frequency (ULF) band which is not considered in this work (49, 50). The limits of these bands could depend on age and we used a method proposed to identify them (50). After we obtained our time series by interpolation [see Behar et al. (50) for details], we divided it into non-overlapping windows of five-minute duration. For each non-overlapping window, we calculated the PSD normalized by the total power. For each normalized PSD, we detected prominent frequency peaks and constructed a histogram of the prominent peak locations (PPL) per individual. We assumed that the histograms were generated from a mixture of Gaussian distributions and used a Gaussian mixture model (GMM) to estimate the Gaussian parameters that best describe the underlying distribution (51). We defined the intersection between consecutive Gaussians as the cutoff frequencies of the band and the upper limit of the HF band (fHFup) as three standard deviations from the Gaussian mean that describes the HF band. We studied the dependence of LF and HF bands on age, analyzing both the cutoff values and the relative power of each band. Since the bands are normalized by total power, the results will only reflect the redistribution of power with age. We called LF/VLF (respectively HF/VLF) to the ratio between the power of the LF band (respectively HF), and that of the VLF band.

The scale relationships between the variables appear as straight lines in log-log plots. In this work, the scale factors were determined by performing a linear regression fit using the least squares method, and the significance of the results was evaluated by an analysis of variance (ANOVA). The size effects can be evaluated either through Cohen's d index or the regression coefficient (R). We used R, and the obtained values indicated that the size effects were negligible in the relationships proposed in this work. We also included the prediction intervals, which is an estimate of a range of values in which a future observation will occur with a certain probability, given what has already been observed.

## 3 Results

**Figure 1:**
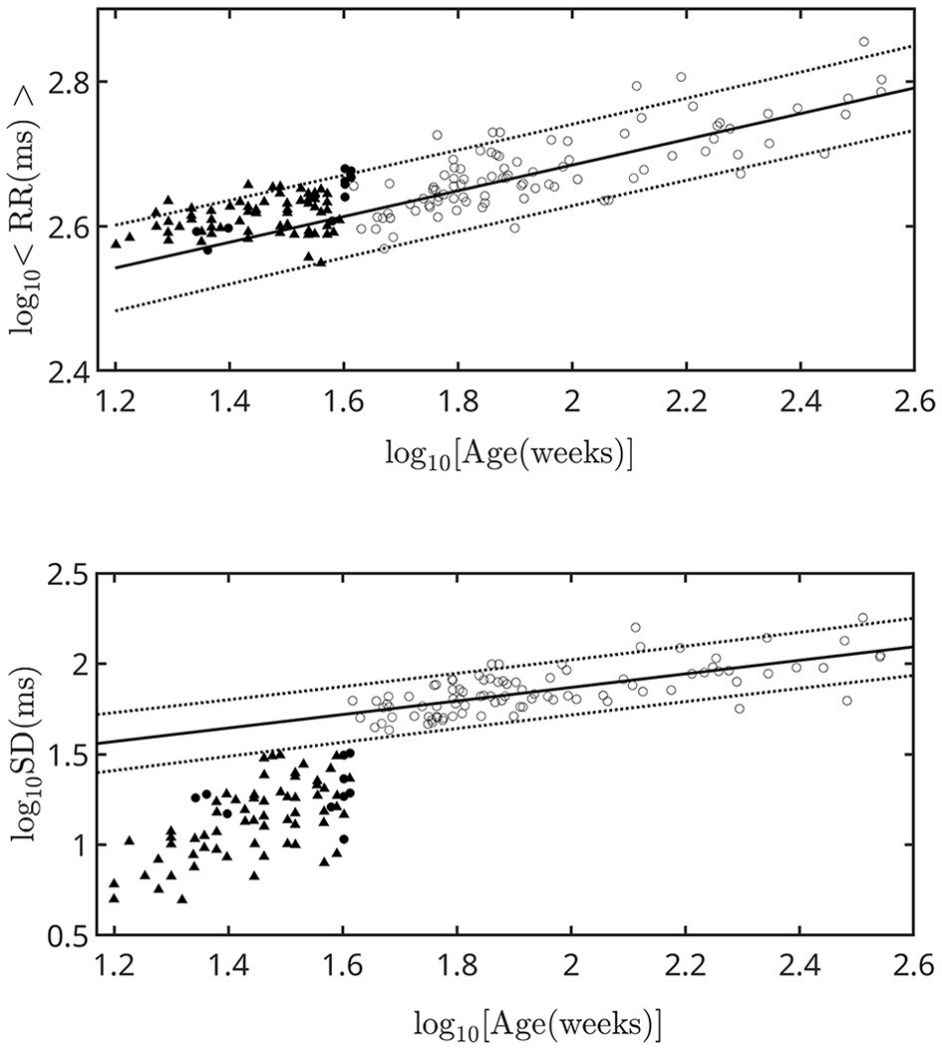
<RR> and SD as a function of age expressed in weeks from conception. The filled symbols correspond to fetuses and the empty symbols to born children. For the data analyzed in this work, the error associated with each point is included within the symbol size. The linear fit was performed on the born children. The thick straight line is the scale law from whose slope the scale factors are calculated. The dotted lines are the limits of the 95% prediction intervals. The triangles indicate the set of values reported in Van Leeuwen et al. (42).

Figure 1 shows the dependence of <RR> and *SD* on age up to puberty. From birth onward we see the previously reported scale behaviors, revealed as linear behaviors after a logarithmic transformation (12). The slopes of the straight lines are the scale factors. Their values (corrected in this work) are 0.15 ± 0.01 (*R* = 0.91, *p* < 10^−4^) and 0.37 ± 0.04 (*R* = 0.84, *p* < 10^−4^) for <RR> and *SD* respectively. In fetuses, <RR> follows the same power law as after birth and throughout life (12). Instead, *SD* has a sudden change at birth. For comparison, we included in Figure 1 data of healthy fetuses obtained by Van Leeuwen et al., by using magnetocardiography (40–42).

**Figure 2:**
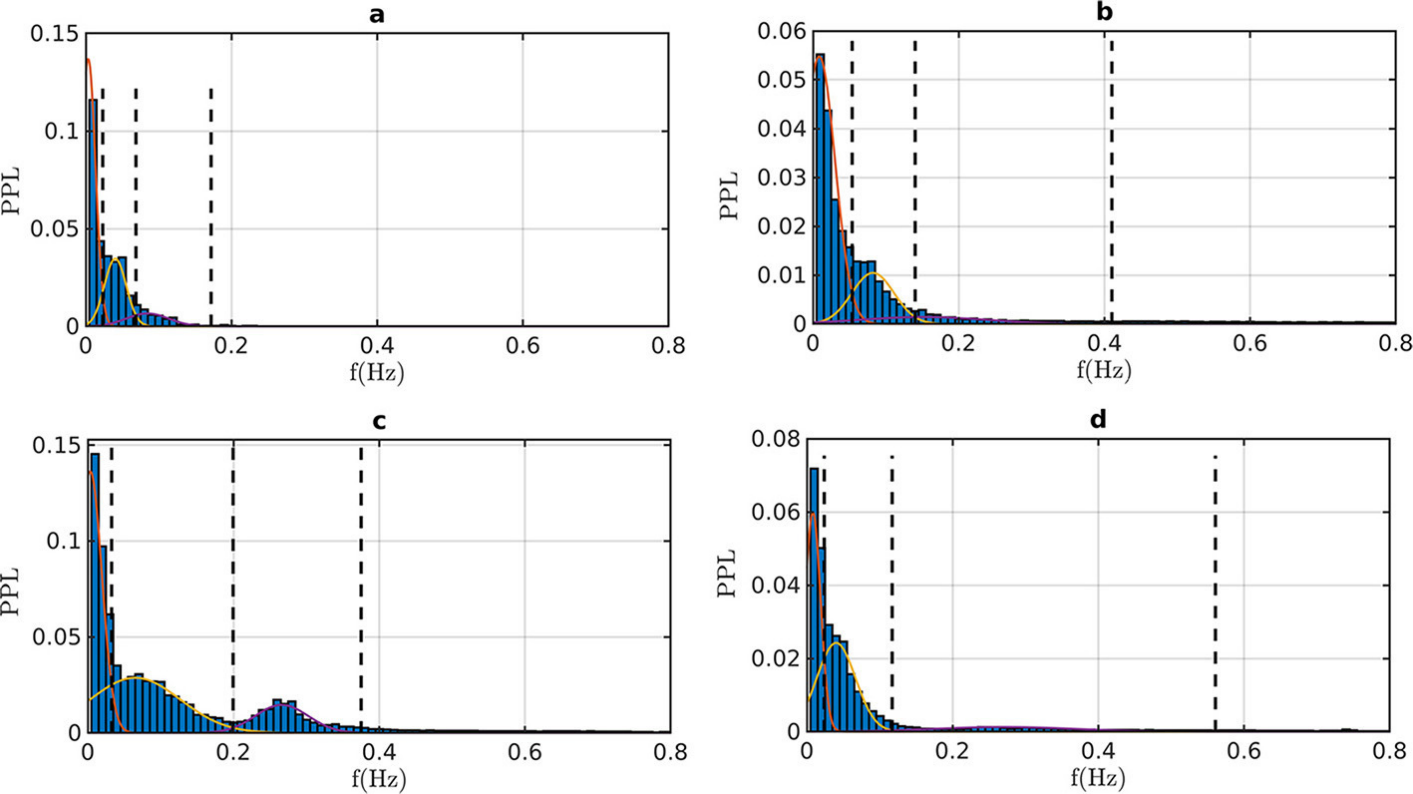
Normalized histograms of prominent peak locations (PPL) of some representative individuals. The thin lines indicate the Gaussian curves, the dashed lines indicate the limits of the VLF, LF, and HF bands. (**A**) Fetuses at 40 weeks of gestation (average of 7 individuals); (**B**) born child aged 44 weeks from conception (1 month from birth); (**C**) healthy adult 28 years of age; **(D)** healthy elderly man 74 years of age.

Figure 2 shows some typical normalized histograms of the prominent peak locations (50). For fetuses, virtually all power is concentrated at low frequencies (*f* < 0.2 Hz). After birth, the power progressively spreads out, only to reconcentrate with aging. The concentration of power is not reflected in the cutoff values of the bands because a broadband background appears, whose origin we will discuss below.

**Figure 3:**
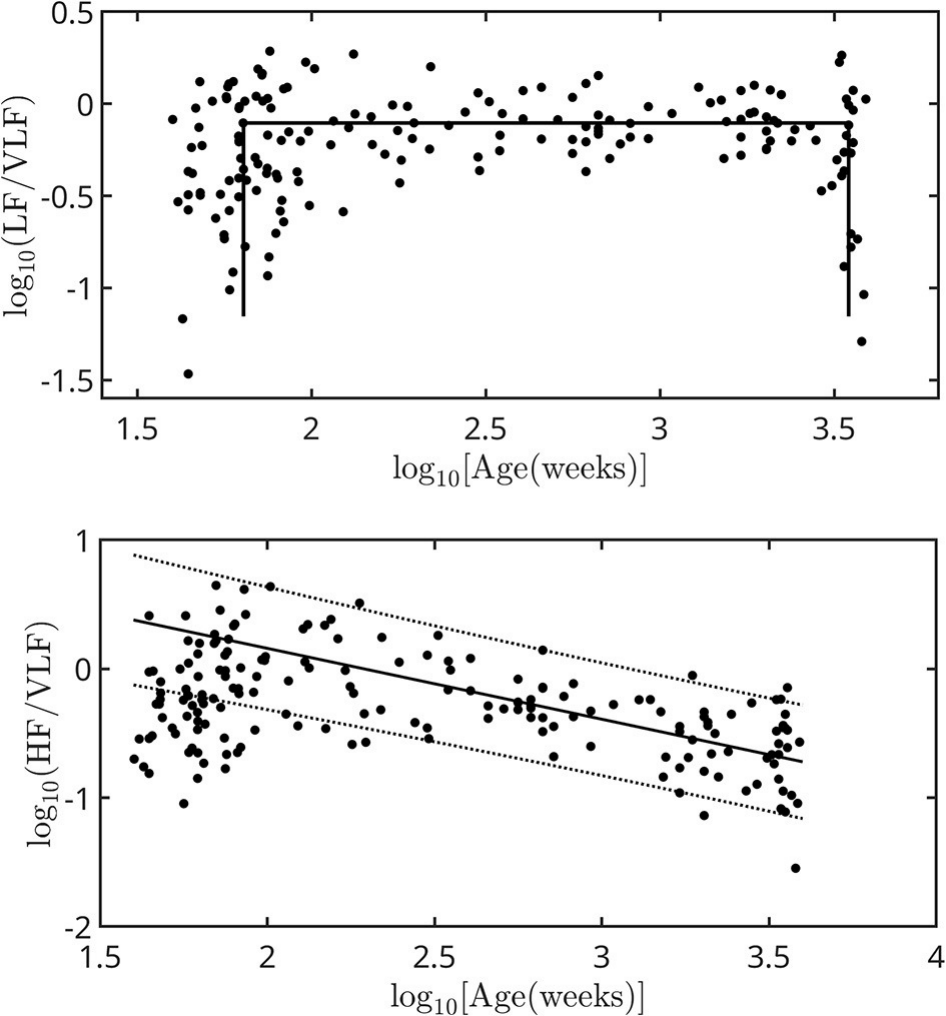
Dependence of LF/VLF and HF/VLF on age expressed in weeks from the moment of conception. The error associated with each point is included within the symbol size. The linear fit was adjusted for *log*_10_[*Age*(*weeks*)]>2. The thick straight line is the scale law from whose slope the scale factor is calculated. The dotted lines are the limits of the 95% prediction intervals.

Figure 3 shows the dependence of LF/VLF and HF/VLF on age. LF/VLF has a square pulse behavior, increasing abruptly during the first 1 or 2 years of life and also decreasing suddenly in old age. HF/VLF has a more moderate behavior; it grows during the first 2 years of life and then decreases following a scaling law given by Equation 2


(2) $$\begin{array}{l} HF/VLF\left(f\right)\alpha x^{-0.55\pm 0.06} \end{array}$$

where x is the age in weeks, *R* = 0.81, and *p* < 10^−3^

**Figure 4:**
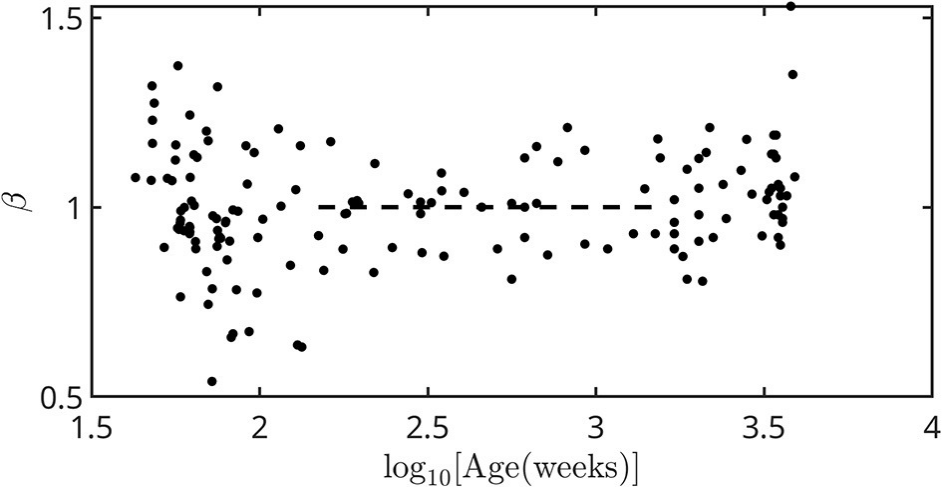
Dependence of β on age expressed in weeks from conception. The dashed line corresponds to the stable value calculated in Garavaglia et al. (12). The error associated with each point is included within the symbol size.

Figure 4 shows the dependence of β on the age, without finite size effects attributable to age rounding. Even so, there are deviations at the extremes of the age range whose origin becomes more evident when we analyze the complete range of frequencies.

**Figure 5:**
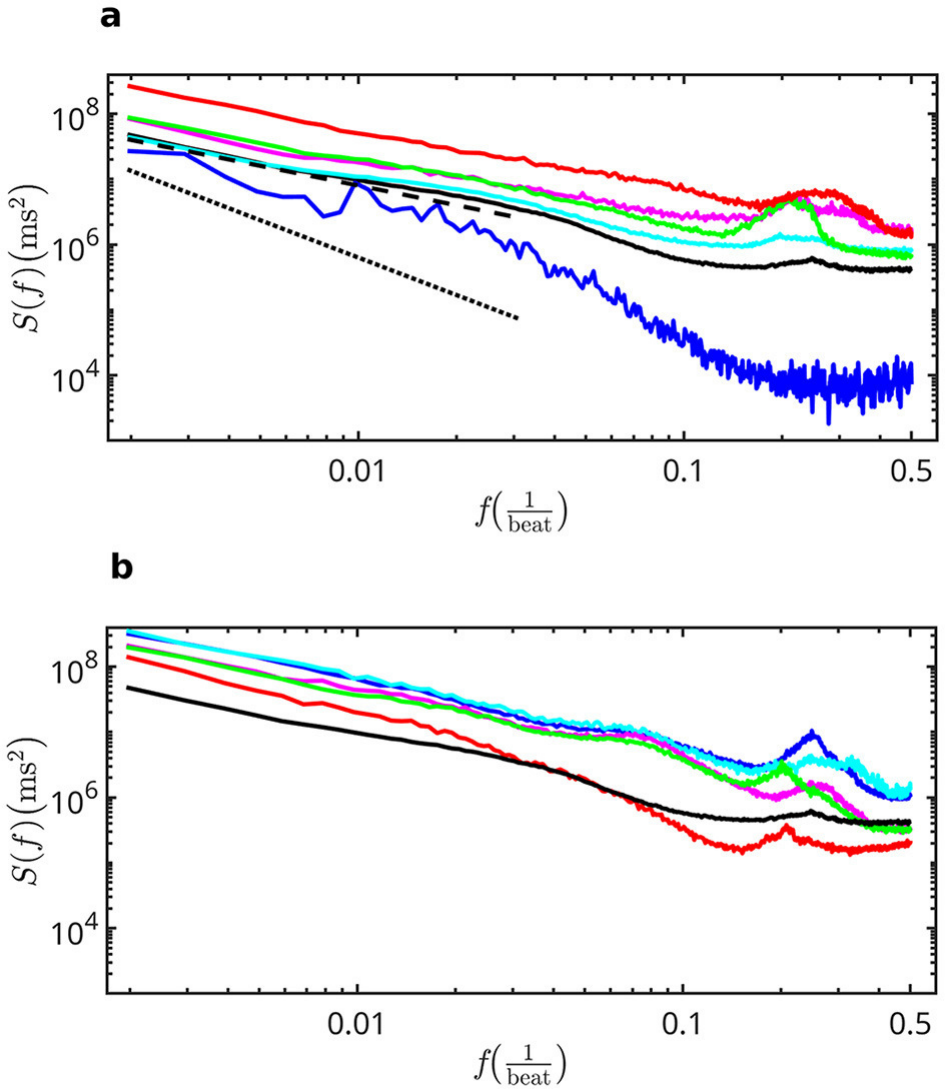
Some averaged S(f) showing representative behaviors at different ages. In **(a)** from bottom to top: Fetuses at 40 weeks of gestation (blue), born children up to 26 weeks (black), born children between 26 and 78 weeks (cyan), born children between 78 weeks and 2 years (magenta), born children between 2 and 4.5 years (green), born children between 7 and 10 years (red). The dotted line has a slope of -1.9 determined in 22-week fetuses. The dashed line has a slope of -1 (pink signal). In **(b)** from top to bottom: children from 11 to 14 years old (blue), young people from 15 to 24 years old (cyan), adults from 26 to 34 years old (magenta), adults from 34 to 45 years old (green), elderly people from 70 to 74 years old (red). The spectrum of children up to 26 weeks (black) was included for comparison.

Figure 5 shows the averaged power spectrum *S*(*f*) at different ages. The HRV signal of fetuses at 40 weeks of gestation is white at high frequencies and pink at low frequencies, with a transition zone at intermediate frequencies. In fetuses at 22 or 23 weeks of gestation, the experimental noise only allowed us to determine the scaling properties at low frequencies, and we found brown signals. Then, during gestation, the signal goes from brown to almost pink in the very low frequency zone. Birth produces significant changes, with the addition of a white contribution across a wide range of frequencies. However, the scaling behavior at low frequencies remains unchanged. The peak of respiratory sinus arrhythmia is also evident in the HF band. From birth onward, the pink signal occupies an increasingly wider band of frequencies, extending from low to intermediate frequencies, and reaching the entire spectral width at puberty, even below the peak of the respiratory sinus arrhythmia. This state remains more or less stable until the age of 35–40. At older ages, the pink signal recedes again to low frequencies. The white contribution also changes with growth, rising slightly and decreasing again to values comparable to those of newborn children.

## 4 Discussion and conclusion

The main achievements of our work were:

(i) We removed rounding effects in the relationships of <RR> and *SD* with age, and corrected scale factors.

(ii) We stated that the scale behavior of <RR> with age is the same from week 22 or 23 of pregnancy and throughout life.

(iii) We determined the existence of an abrupt change in *SD* at birth and the appearance of a white contribution, which increased slightly with growth and decreased during aging.

(iv) While the LF and VLF bands behaved similarly with age, except in the first and last years of life, the HF/VLF ratio increased during the first 2 years of life and then decreased following a scale law determined in this work (Figure 3).

(v) The low-frequency behavior of the HRV signal went from brown to pink during pregnancy. This result was in agreement with the increase in *SD* shown in Figure 1.

(vi) The scale factor β changed during the first years of life, then settling at a constant value until adulthood (Figure 4).

(vii) The frequency range in which the HRV behaved as a non-white colored signal began at low frequencies, then expanded toward the intermediate ones reaching the entire spectral width. This state remained more or less stable from 11–14 years to 35–40 years of age. At older ages, the colored signal receded again to low frequencies.

Van Leeuwen et al. reported a positive correlation of <RR> with gestational age, and this result is in agreement with the scaling behavior that we reported in the present work. Finite-size effects are evident, which decrease the slope value. Eliminating them requires knowing the gestational age within a few days, and we are working on this further. Van Leeuwen et al. found a positive correlation of SD with gestational age. The data did not follow the scale behavior reported for born individuals. Hoyer et al. also studied the HRV of 294 healthy singleton fetuses by using magnetocardiography (52). They reported SD values between weeks 21 and 42 of gestation, which are all significantly lower than those reported by Van Leeuwen et al. Mølgaard et al. studied the fetal heart rate variability using time-domain and spectral analysis. Their data are compatible with those reported in this work (38).

Yaniv et al. conducted studies in mammals, including humans, to elucidate the contributions of SAN and ANS. They analyzed the differences in the HRV between basal and ANS blockade conditions (20, 21), and studied the dynamics of denervated SAN tissue isolated from mice and rabbits (22). They stated that the main features of the contribution of ANS in adult humans are short-term time-domain HRV metrics, location of frequency peaks in the LF and HF bands, spectral power in these bands, and DFA base level (or flat power spectrum). They also suggested modeling the contribution of the ANS as a stochastic process with white noise characteristics. Our findings in humans support this hypothesis. In our data, *SD* underwent an abrupt change during birth, and the analysis of the power spectra revealed the addition of a white component at birth, which evolved only slightly during growth, and decreased in old age. This white contribution had slight daily variations but was always noticeable in born individuals. We also found that the LF/VLF ratio exhibited a roughly square profile with abrupt changes at the extremes of life. This suggests that both bands behave very similarly throughout the human lifetime. In contrast, the HF/VLF ratio increased during the first 2 years of life and then decreased following a scale law until old age. This result is consistent with the findings mentioned above.

Yaniv et al. found that the SAN contributes mainly to long-range patterns and very low frequencies within the beat intervals. The primary spectral contribution of the SAN is in the VLF band, within the so-called “invariant” region in the normalized PSD. They concluded that the rich structure embedded in the beat interval signal originates in the SAN. Following this reasoning, we studied the scale behavior of the power spectrum as a function of age.

It is known that the scale factor of the power spectrum takes a value of β = 1 in healthy young adults, and that is why HRV is said to have a pink signal structure. This work revealed that the scale behavior actually begins at low frequencies, in the VLF band. Besides, β depends on age (12), and in fetuses it evolves during pregnancy. This work revealed that the value of β decreases from ~ 2 (brown signal) as the pregnancy progresses. Birth does not causes an abrupt change in β; its value continues to change until ~ 2 years of age and then stabilizes at the known value until adulthood. This evolution can be observed in Figure 4 and is present even after removing finite size effects due to rounding errors. From the analysis of the power spectra in Figure 5, it is observed that the scale behavior spreads with growth toward higher frequencies. At some age between 11 and 14 years, it occupies the entire frequency space, even in the HF band where the respiratory peak simply overlaps with it. Around these ages, *SD* reaches its maximum value. At old age, the high-frequency background which had been established at birth and increased slightly throughout life, begins to decline. The scale behavior also recedes, moving again toward the low frequency zone. Finally, β starts to increase again toward a brown behavior.

Yaniv et al. studied the effect of aging on HRV in mammals: (i) in the basal state *in vivo*; (ii) during a state of intrinsic autonomous denervation *in vivo*; and (iii) *ex vivo*, in isolated intact SAN tissue, in which the autonomic neural input is absent. They demonstrated the high variability of the firing rate of isolated SAN cells and how this behavior changes when considering a small SAN tissue or a completely denervated heart. Rose et al. reached similar conclusions by performing studies: *in vivo* (with and without ANS blockade), in isolated atrial preparations, and in isolated SAN myocytes (53). The authors performed a multifractal analysis and concluded that the firing rate of SAN myocytes is random. However the complexity increases as the network of cells becomes more structured. These findings are consistent with the idea that the complexity in HRV is related to the structure (and the size) of the SAN. Likewise, complete blockade of the ANS results in an almost monofractal behavior. The authors concluded that the HRV signal in healthy mice progressively increases levels of organization from isolated cells to intact mice.

Based on this evidence, we suggest that the complex structure of the HRV mainly reflects the structural complexity of the SAN, which continuously evolves from the fifth month of gestation and increases until reaching its maximum expression at puberty. There is a white noise component in the HRV that begins at birth and can be attributed to the ANS. Thus, the SAN, evolving structurally from its appearance in the fifth month of gestation and throughout life, could be responsible for the modulation of the neural stimulation provided by the ANS. In aging, a deterioration of both the SAN structure and neuronal stimulation, occurs.

Parasympathetic nervous system blockade experiments in mice resulted in an increase in HRV randomness. The results support the influence of SAN architecture on HRV because the block occurs at the neuromuscular junction. Consistently, the authors concluded that intrinsic SAN activity does not fully explain the complexity of HRV (53).

The scale behaviors of <RR> and *SD* with age strongly support the existence of a main mechanism driving the entire process. A recent review highlights the role of fibrosis in the normal function of the SAN (24). Interstitial fibrosis is inherent to the SAN and can be detected in the auricular tissue from the fifth month of gestation. Furthermore, a correlation has been established between aging and increased fibrotic content in the SAN, which is strongly associated with the deceleration of electrical conduction.

Our results encourage the study of the role of fibrosis in the establishment and deterioration of the SAN structure, and neuronal stimulation in the healthy heart. To the authors' knowledge, a relationship between fibrotic content in the healthy heart and fractal or multifractal characteristics of HRV has not been established yet. We hope our work will stimulate research into this relationship. Antifibrotic approaches could present promising therapeutic options in the future.

In summary, focusing on the characteristics of the HRV attributed to the SAN and ANS, and their evolution with age in healthy individuals, we support the idea that the structure of the SAN modulates the neural input of the ANS. This structure evolves during pregnancy, growth and aging, and the HRV reflects these changes.

The main limitations of our work are found in the study of fetal HRV series. Changes in fetal heart rate and its variability during gestation are well documented and our results are consistent with previous findings (40–42, 52, 54). However, longer and earlier studies are desirable and we have work in progress in this regard.

## Data Availability

The original contributions presented in the study are included in the article/supplementary material, further inquiries can be directed to the corresponding author.
